# P-2052. Pre-existing Humoral Immunity to Seasonal Coronaviruses and Effect on SARS-CoV-2 Antibody Responses on SARS-CoV-2 Vaccination and Infection

**DOI:** 10.1093/ofid/ofae631.2208

**Published:** 2025-01-29

**Authors:** Etsuro Nanishi, Matthew K Hwang, Walter Byrne, Kimberly Thompson, Nicole Wisener, Julia Upton, Aaron Campigotto, Maria Rosa La Neve, Jasmik S Saini, Alice Litosh, Ana Citlali Marquez, Agatha N Jassem, Upton D Allen

**Affiliations:** The Hospital for Sick Children, Toronto, ON, Canada; The Hospital for SickKids, Toronto, Ontario, Canada; Hospital for Sick Children, Toronto, Ontario, Canada; The Hospital for Sick Children, Toronto, ON, Canada; The Hospital for Sick Children, Toronto, ON, Canada; Hospital for Sick Children, Toronto, Ontario, Canada; Hospital for Sick Children, Toronto, Ontario, Canada; The Hospital for Sick Children, Toronto, ON, Canada; The Hospital for Sick Children, Toronto, ON, Canada; Hospital for Sick Children, Toronto, Ontario, Canada; BC Centre for Disease Control, Vancouver, British Columbia, Canada; University of British Columbia, Vancouver, British Columbia, Canada; Hospital Sick Children, Toronto, Ontario, Canada

## Abstract

**Background:**

High homology and potential cross-reactive immune responses between SARS-CoV-2 and seasonal human coronaviruses (HCoVs) have been described by several studies. However, the role of pre-existing immunity to HCoVs in the outcome of SARS-CoV-2 infection and vaccination is still unclear.

Anti-spike IgG titers against human coronavirus (HCoV)-229E, -HKU1, -NL63, and -OC43 by age of participants

**Figure:**
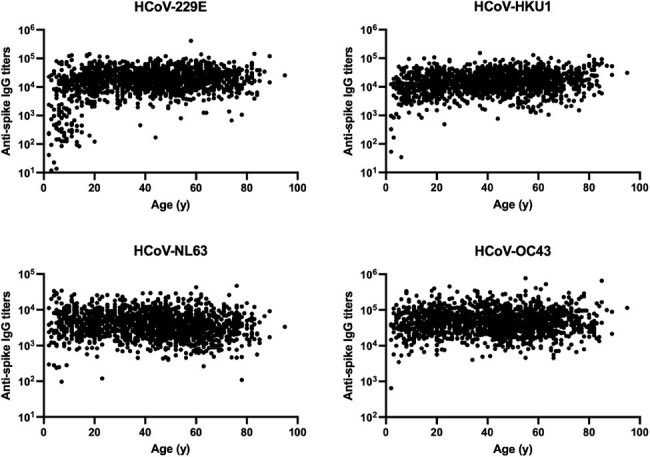

N=1,675 serum samples were collected from participants aged 2 to 95 years (median 44). anti-spike IgG titers against HCoV-229E, -HKU1, -NL63, and -OC43 were quantified by electrochemiluminescent immunoassay. Each dot represents the anti-HCoV spike IgG titer and age of the participant.

**Methods:**

Pediatric and adult participants were enrolled. We collected demographic data and vaccination status, as well as serum samples at a single time point between August 2020 and August 2023. Anti-spike IgG titers against SARS-CoV-2 and HCoV-229E, -HKU1, -NL63, and -OC43 were quantified by electrochemiluminescent immunoassay. SARS-CoV-2 infection status was determined by the presence of anti-SARS-CoV-2 nucleocapsid antibodies. Correlations were assessed by two-sided Spearman rank-correlation tests.

Correlations between SARS-CoV-2 and hCoV-229E, -HKU1, -NL63, and -OC43 spike IgG titers among SARS-CoV-2 unvaccinated participants

**Figure:**
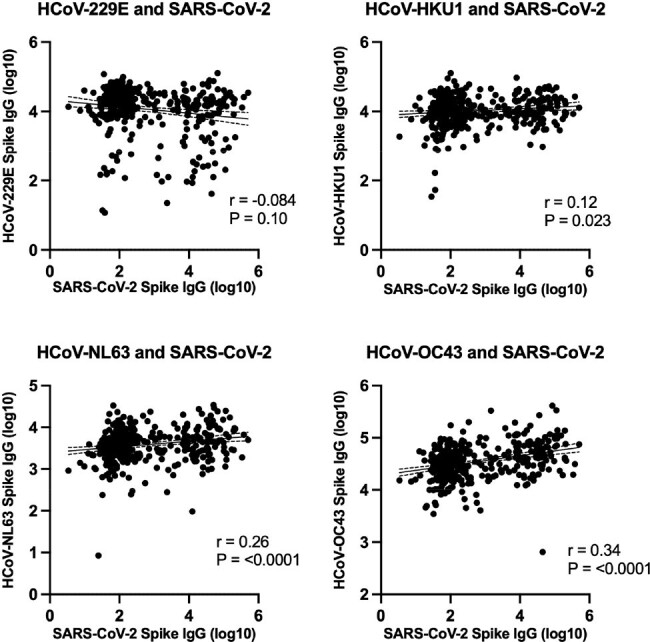

To evaluate the effect of immunity against HCoVs on SARS-CoV-2 infection, correlations between SARS-CoV-2 and HCoV-229E, -HKU1, -NL63, and -OC43 spike IgG titers among SARS-CoV-2 unvaccinated participants are shown (N=380). Each dot represents individual participants. Solid and dotted lines respectively indicate linear regression and 95% confidence interval. Correlations were assessed by two-sided Spearman rank-correlation tests.

**Results:**

Sera were collected from N=1,675 participants of which 5.7% were ≤10 years (age range: 2-95 yrs, median: 44 yrs). HCoV titers rapidly increased in early childhood and the majority of adults had immunity against HCoVs. We first analyzed N=380 sera from SARS-CoV-2 unvaccinated participants. SARS-CoV-2 titers positively correlated with HCoV-OC43, -HKU1, and -NL63 titers. Furthermore, HCoV-OC43 titers were significantly higher in participants post-SARS-CoV-2 infection, determined by the presence of SARS-CoV-2 nucleocapsid antibodies, as compared to non-infected participants (geometric mean, 49,256 vs 29,613; *P*< 0.01). Next, to evaluate the correlation between HCoV immunity and SARS-CoV-2 titers on vaccination, sera from N=1,059 SARS-CoV-2 non-infected participants were analyzed. Notably, positive correlations between SARS-CoV-2 and HCoV-OC43, and -HKU1 anti-spike IgG titers were demonstrated (r=0.43 and 0.27; both P< 0.0001). Although higher numbers of SARS-CoV-2 vaccinations were associated with more SARS-CoV-2 antibodies, HCoV-OC43 titers did not show a correlation.

Correlations between SARS-CoV-2 and hCoV-229E, -HKU1, -NL63, and -OC43 spike IgG titers among SARS-CoV-2 non-infected participants

**Figure:**
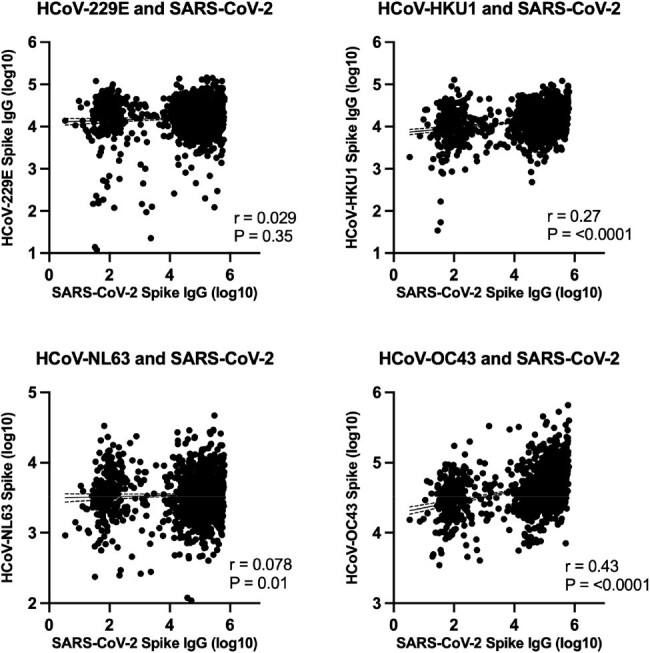

To evaluate the effect of immunity against HCoVs on SARS-CoV-2 vaccination, correlations between SARS-CoV-2 and HCoV-229E, -HKU1, -NL63, and -OC43 spike IgG titers among SARS-CoV-2 non-infected participants, determined by the absence of SARS-CoV-2 nucleocapsid antibodies, are shown (N=1,059). Each dot represents individual participants. Solid and dotted lines respectively indicate linear regression and 95% confidence intervals. Correlations were assessed by two-sided Spearman rank-correlation tests.

**Conclusion:**

HCoVs immunity was acquired in early childhood. HCoV-OC43 titers were higher in SARS-CoV-2 infected participants compared to non-infected. In SARS-CoV-2 non-infected participants, positive correlations were seen between SARS-CoV-2 and HCoV-OC43 and -HKU1 titers. Our data indicates that immunity against HCoVs may enhance SARS-CoV-2 immune responses on infection and vaccination.

Anti-spike IgG titers against SARS-CoV-2 and HCoVs by numbers of previous SARS-CoV-2 vaccines

**Figure:**
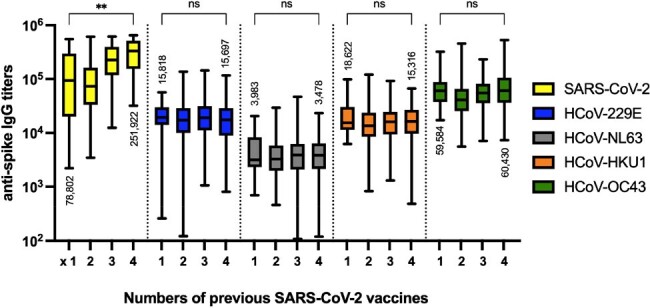

SARS-CoV-2 and HCoV-229E, -HKU1, -NL63, and -OC43 spike IgG titers among SARS-CoV-2 non-infected participants, determined by the absence of SARS-CoV-2 nucleocapsid antibodies, are shown by numbers of previous SARS-CoV-2 vaccination (N=780). Geometric mean titers are listed. Data were analyzed by Kruskal–Wallis test. ** P<0.01.

**Disclosures:**

Julia Upton, MD, ALK Abello: Advisor/Consultant|ALK Abello: Grant/Research Support|Bausch Health: Advisor/Consultant|DBV Technologies: Grant/Research Support|Pfizer: Advisor/Consultant|Pharming: Advisor/Consultant|Regeneron: Grant/Research Support|Sanofi: Grant/Research Support

